# Body Packing: From Seizures to Laparotomy

**DOI:** 10.1155/2015/208047

**Published:** 2015-03-26

**Authors:** Joanna M. Janczak, Ulrich Beutner, Karin Hasler

**Affiliations:** ^1^Department of General, Visceral and Transplantation Surgery, Kantonsspital St. Gallen, Rorschacherstrasse 95, 9007 St. Gallen, Switzerland; ^2^Emergency Department, Kantonsspital St. Gallen, Rorschacherstrasse 95, 9007 St. Gallen, Switzerland

## Abstract

Body packing is a common method for illegal drug trafficking. Complications associated with body packing can be severe and even lead to rapid death. Thus, a timely diagnosis is warranted. As most body packers initially do not show any symptoms, making a correct diagnosis can be rather challenging. We describe a case of a 41-year-old male, who was admitted with an epileptic seizure and who turned out to be a cocaine intoxicated body packer. Due to neurological and cardiovascular deterioration an emergency surgery was performed. Four bags of cocaine could be removed. We discuss the current management regimen in symptomatic and asymptomatic body packers and highlight pearls and pitfalls with diagnosis and treatment.

## 1. Introduction

Body packing refers to the practice of swallowing illegal drugs in small containers (typically plastic bags or condoms) for intestinal transport across country borders. Only a few percent of the body packers show clinical symptoms [1], but they can be associated with a broad spectrum of gastrointestinal, neurological, or cardiovascular complaints. Clinical manifestation depends on many factors such as type and amount of the drug, nature of container, duration of retention, grade of rupture, location in the gastrointestinal tract, or the general health of the courier. Diagnostic uncertainty and the lack of a medical history due to the patient's inability and unwillingness to collaborate make an accurate diagnosis in a timely fashion very challenging. Furthermore, radiological exams and laboratory tests are often inconclusive in these cases.

## 2. Case Description

**Figure 1:**
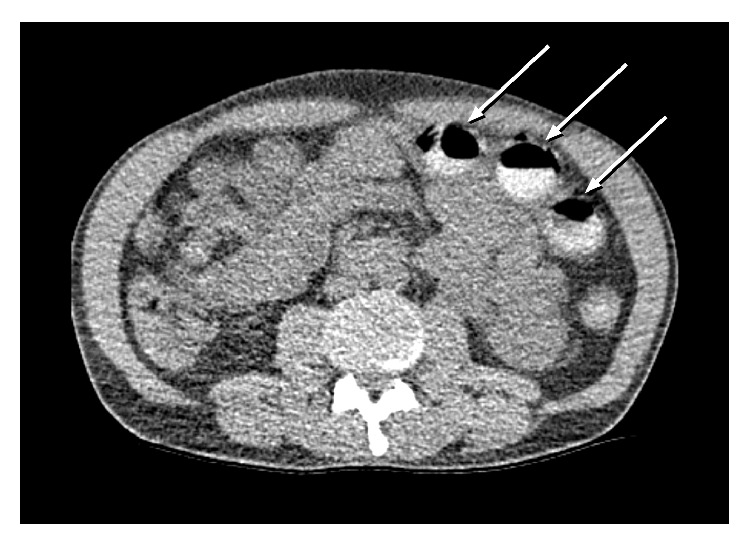
CT scan showing three of four, round, foreign bodies (arrows) in the left transverse colon.

A confused 41-year-old male was found in a park and brought to emergency department by the paramedics. During transit he had two generalized seizures. On the admission the patient appeared confused but conscious with a Glasgow Coma Scale of 13. He behaved aggressively and spoke neither English nor a local language. An interpreter was consulted, but the patient showed aggressive behaviour, therefore making it impossible to take a history. The physical examination was unremarkable except for a tongue bite and several bruises and scratches over the whole body. The pupils were moderately dilated and sluggishly reactive. All vital signs were stable (heart rate: 108 beats per minute, blood pressure: 123/80 mmHg, oxygen saturation: 98% at room air with a normal respiratory rate, and a body core temperature of 37.2°C). Laboratory tests revealed a leukocytosis of 25.1 G/L, elevated values for C-reactive protein (12 mg/L), creatinine kinase (2185 U/L), myoglobin (618 *μ*g/L), creatinine (143 mmol/L), and uric acid (1344 mmol/L) suggesting renal insufficiency. Electrolytes, liver enzymes, troponin I, coagulation, and haematology were in the normal range. An electrocardiogram showed a sinus tachycardia. Neurology service was consulted because of ongoing confusion and for assessment of epilepsy. A cranial CT scan ruled out haemorrhage or other focal lesions. Lorazepam (2 mg, IV) was administered twice with no effect on his neurological status. The patient started to sweat and became hypertensive (systolic blood pressure of 180 mmHg) and more tachycardic (heart rate of 140/min). A drug screening (Triage 8) was performed, which was positive for cocaine. Finally he admitted the cocaine consumption but refused to give further information. Benzodiazepines as well as phentolamine were given. In order to further investigate the drug intoxication a plain abdominal dual-energy CT scan was requested. Four round, hyperdense, foreign bodies were found in the transverse colon (Figure 1).

**Figure 2:**
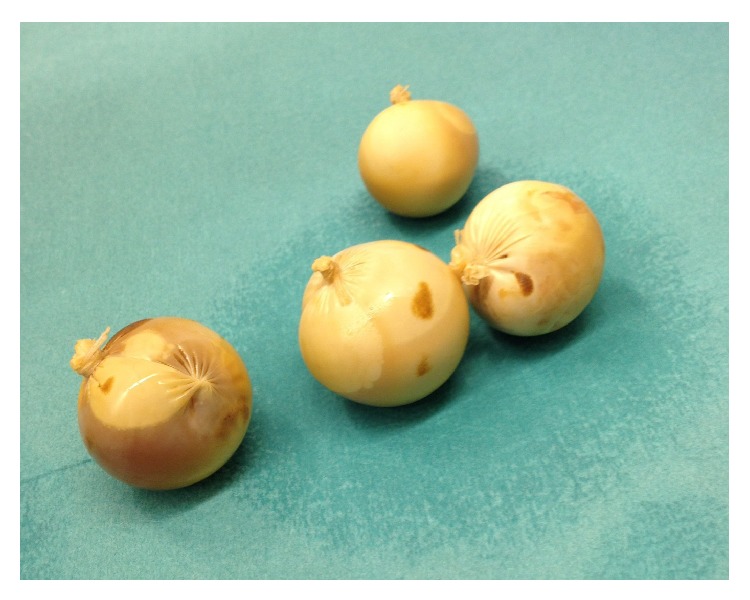
Foreign bodies removed from the colon.

An emergency laparotomy was indicated and carried out immediately. Following colotomy four packages (Figure 2) were removed. Thorough exploration revealed no further packages. The procedure took 60 minutes and postoperatively the patient was on observation for 24 h in the intensive care unit. The neurological signs as well as the renal insufficiency improved rapidly under conservative therapy. The further clinical course was unremarkable, and the patient was discharged six days after surgery.

## 3. Discussion

Internal concealment of illegal drugs is increasingly seen even outside bigger cities without international airports. Clinical symptoms due to complications associated with body packing are rare as 1.4 to 6.6% [2–5] probably owing to improvements in drug wrapping [6]. However, it must be highlighted that mortality can be as high as 56%, when symptoms occur [6]. Typical cardiovascular complications such as tachycardia, ventricular fibrillation, hypertension, myocardial infarction, or even cardiac arrest occur in around 75% of body packers. Neurological signs comprise anxiety, seizures, or altered consciousness often associated with agitation and anxiety as well as coma. Gastrointestinal symptoms are mostly related to bowel obstruction occurring in 25% of cases [7]. Drug container rupture leads to rapid intestinal drug absorption with possible fatal consequences [3]. Interestingly, the number of drugs packages does not correlate with the rate of perforation [8]. The reported rate of surgical removal of drug bags due to failure of spontaneous intestinal passage is up to 5% [8]. Often patient's history is unreliable and the diagnosis of body packing is solely based on the physician's intuition. Physical examination, radiological findings, and laboratory test are mandatory in confirming or rejecting the suspected diagnosis. Physical examination should begin with the classical ABCDE survey. Although Beckley et al. [3] found that physical examination was unremarkable in 81% of the cases, a thorough neurological, abdominal, and rectal examination preceding diagnostic imaging is crucial [9]. General laboratory work including urea, electrolytes, liver enzymes as well as coagulation, and haematology should be obtained. We also performed a 12-lead ECG to detect arrhythmias or myocardial ischemia.

Plain radiography and contrast enhanced computer tomography are recommended radiological examinations. An abdominal CT scan is preferable due to the better specificity and sensitivity, especially for the detection of liquid cocaine, which is practically invisible on normal X-ray images [3, 4]. Sensitivity of plain abdominal X-rays, however, can be as low as 40% [9]. Current drug packaging made of nonradiopaque materials is generally difficult to detect radiologically [10]. False positive results were observed because of bladder stones, other calcifications, or coprostasis [8]. Low-dose CT seems to be an effective alternative to abdominal radiography [11].

Urine tests for rapid drug screening can be useful in symptomatic patients but yield false negative results in 48% of asymptomatic patients [12]. While some authors oppose urine drug tests due to their poor sensitivity of 37% [8], other authors report a sensitivity of up to 96.3% and a specificity of up to 99.8% and recommend routine use prior to radiological examinations [6].

Asymptomatic body packers should be monitored closely, preferably on an intermediate or intensive care unit, allowing for a quick response in case of complications or clinical deterioration. Asymptomatic patients should be started on activated charcoal, which reduces the lethality in oral cocaine intoxication [8]. Bowel irrigation with polyethylene glycol can be used to induce purging of the body bags [8, 13, 14].

Oily laxatives should not be applied due to the high risk of perforating the latex wrapping [8, 14]. The recommended observation time in case of extended intestinal passage varies between 72 hours and 7 days [2, 3].

One should not attempt to remove the drug containers endoscopically as this can result in ruptures [2, 8]. However, some successful gastroscopic bag recoveries from the upper gastrointestinal tract were reported [2, 8]. In the study of Schaper et al. [4] the average length of hospital stay ranged from 2.8 days for conservatively treated body packers to 10.4 days for surgically treated body packers.

Indications for surgery are signs of intoxication, bowel obstruction, and extended intestinal passage (over 48 hours and suspected leakage) [1, 2]. Immediate surgery is vital in these patients. In the study of Schaper et al. [4], only 32% of the symptomatic patients survived until the operation, and the majority died before the intervention could begin. This holds true in cases of cocaine intoxication especially, since no antidote is available [5, 7]. A relative indication for surgery is failure of intestinal passage for more than five days according to Silverberg et al. [10].

Other rare emergency situations were gastric outlet syndrome, gastrointestinal ulceration, or bleeding as well as respiratory arrest due to airway obstruction by the containers [10]. Whether preemptive surgery should be performed in asymptomatic body packers is still under debate. A prophylactic operation was recommended few years ago for unsophisticated drug containers due to the high risk of rupture [8].

According to Bogusz et al. [12] a preemptive surgery should be reserved for symptomatic patients because in asymptomatic patients the risks related to surgery outweigh the risks of conservative therapy. Intraoperative morbidity and mortality were estimated to be up to 16% and 2%, respectively [2]. The preferred surgical approach for bag retrieval is by enterotomy. The number of enterotomies correlates with the surgical site infection rate (up to 40%) [10]. If the bags are located more distally in the colon or in the rectum, they can be pushed towards and through the anus without performing an enterotomy [2, 10].

Radiological imaging should be repeated after the operation to document removal of all containers [8].

## 4. Conclusion

Diagnostic uncertainty related to the lack of a good medical history as well as the diversity of clinical symptoms challenges the clinical management of body packers. It is generally advisable to perform an abdominal CT scan while urine drug tests seem to be less reliable in confirming the suspected diagnosis. Although most body packers remain asymptomatic, a close monitoring is crucial. Clinical deterioration can be sudden and requires immediate laparotomy and enterotomy. As long as there are no official guidelines for the clinical management of body packers, every hospital should set up its own diagnostic and treatment algorithms, since any delay in treatment can be fatal.
